# ZHENG May Contribute to Obesity Phenotypes Based on Body Composition: A Pilot Study on the Traditional Chinese Medicine Approach

**DOI:** 10.1155/2014/580803

**Published:** 2014-03-10

**Authors:** Feng Tao, Hao Lu, Jean-Michel Oppert, Arnaud Basdevant

**Affiliations:** ^1^Shanghai Key Laboratory of Traditional Chinese Clinical Medicine, Department of Endocrinology & Metabolism, ShuGuang Affiliated to Shanghai University of Traditional Chinese Medicine, No. 528 Road ZhangHeng, Shanghai 201203, China; ^2^Department of Nutrition, Heart and Metabolism Division, Pitié-Salpêtrière University Hospital, No. 47-83 boulevard de l'Hôpital, 75013 Paris, France; ^3^CRNH-Ile de France, Institute of Cardiometabolism and Nutrition (ICAN), University Pierre et Marie Curie, No. 47-83 boulevard de l'Hôpital, 75013 Paris, France

## Abstract

*Objective.* Obesity therapy needs new approaches to complement current phenotyping systems. This study aims to assess associations between the Traditional Chinese Medicine (TCM) ZHENG and obesity phenotypes. *Methods*. We assessed medical history and habitual physical activity and measured body composition, fasting plasma glucose and insulin, and lipids. We collected TCM data through face-to-face interview. ZHENG elements (essentials and locations) were identified by TCM practitioner. Primary ZHENG was assessed by cluster analysis. *Results.* In 140 consecutive subjects enrolled in a university clinic (body mass index (BMI): 39.9 ± 5.8 kg/m2), ZHENG essentials were identified as “QiXu,” “Re,” “YinXu,” and “TanShi” (totally 86.8%). Locations were “Shen,” “Wei,” “Pi,” and “Gan” (totally 91.8%). Four types of primary ZHENG were identified: A (37.1% of subjects), B (16.5%), C (35.7%), and D (10.7%). Subjects in type D showed elevated BMI, total fat mass (FM), FM index, trunk FM, and less physical activity, as compared with others. Subjects in type B changed regional body composition (reduced trunk FM% and elevated appendicular FM%). Biological parameters did not differ across primary ZHENG clusters. *Conclusions*. Obesity phenotypes based on body composition differ according to ZHENG in obese patients. This study is a first step toward understanding the contribution of TCM to obesity phenotyping.

## 1. Introduction

There is a global increase in obesity prevalence worldwide including countries undergoing rapid economic development, such as China and India [1]. A Chinese nutrition survey shows that the prevalence of overweight and obesity was 19.2% and 15.0%, respectively [2]. The medico-economic impact is significant because of its close associations with diabetes, hypertension, cardiovascular disease, respiratory disturbances, and certain cancers [3, 4]. The effectiveness of obesity management is a key issue. Therapeutic progress relies on identifications of new therapeutic targets, developments of new pharmacological agents, and advanced knowledge of basic mechanisms leading to obesity and its complications. All of the above depend on a better phenotypic characterization of an extreme heterogeneousness condition in both its origins and consequences.

Obesity is defined as an abnormal or excessive accumulation of body fat that may impair health. Body fat content and its relation to ill health are therefore central to the definition and understanding of obesity phenotypes [5]. Many phenotypes can be identified; however, neither phenotype is completely satisfactory. For instance, body mass index (BMI), which forms the basis for the definition of obesity, can be considered as “poor” at individual level because it does not provide specific guidance on body composition. Furthermore, cut-offs commonly used do not characterize changes in fat mass which may appear by age, sex, ethnic groups, or sports training. Total fat mass (FM) is considered as a primary phenotype; however, assessing FM accurately remains difficult and expensive in clinic or large populations. Moreover, there are no established reference data and associations with health risks are less documented. Waist-hip ratio is simple to assess body fat distribution but is considered as cumbersome by many practitioners. Bioimpedance analysis and dual-energy X-ray absorptiometry (DXA) present with shortcomings and limitations for measuring body fat distribution [6]. From the point of view of clinical practice, new phenotypes are expected. We need new approaches to complement and improve our current framework for assessing obesity [7].

Traditional Chinese Medicine (TCM) is a major representative of complementary and alternative medicine. Its efficacy has been proved in chronic diseases, including obesity [8–10]. ZHENG (*in *Chinese, also known as syndrome or pattern) is the key concept, identified from a comprehensive analysis of clinical information through TCM methods, like observation, inquiring patient, and tongue and pulse analyses [11]. ZHENG can be regarded as a summary of location, etiology, pathology, and trend in certain stages of disease, also as a synthesis of disease effect and body reflection. Almost all the TCM therapeutic methods are based on ZHENG. As a kind of combined plane of linear connection of various syndromes, it was hard to comprehend for one person without TCM culture education. Recently, Chinese researchers proposed to divide ZHENG into elements, including “essentials” and “locations.” “Essentials” and “locations” are two categories of ZHENG elements, which can be understood as what kinds of pathological changes and where these changes exist. By this way, ZHENG can be regarded as a system with multiple dimensions including essentials and locations. This maneuverable method has wide application in recent integrative medicine research [12].

Up to now, ZHENG-related studies have shown certain achievements in integration with western medicine [13]. ZHENG has been explored in the context of neuroendocrine-immune networks [14]. Other studies showed imbalanced network biomarkers according to ZHENG categories (e.g., for its representative tongue coating) [15, 16]. ZHENG could improve efficacy of selected biomedical intervention and build up a molecular network in rheumatoid arthritis [17–19]. By this way, ZHENG could provide a new framework for disease phenotyping from an alternate medicine viewpoint.

In the past two decades, work by Chinese researchers has provided evidence of some links between ZHENG and obesity phenotypes. Chinese obese patients (defined as BMI > 28 kg/m^2^) with ZHENG “Pi-Wei-Re” or “Pi-QiXu” were shown to have increased BMI compared to other patients; one study in 874 Chinese subjects showed that ZHENG essential “Re” (interior heat) and “YinXu” (Yin deficiency) were closely related hyperglycemia associated with obesity; another study showed that subjects with ZHENG essential “TanShi” (phlegm-dampness obstruction) had increased waist circumference and waist-hip ratio (as measures for abdominal obesity) by comparison to non-“TanShi” subjects [20–22]. These studies were carried out in China and data only included anthropometric measures such as BMI and circumferences. There is therefore a need for further research based on more direct body fat phenotypes. In this study, we investigated ZHENG feature (including essential, locations, and primary ZHENG) in French obese patients and we assessed relationships of ZHENG to body composition and associated obesity phenotypes.

## 2. Materials and Methods

### 2.1. Subjects and Protocol

The study population included patients admitted consecutively over a 6-month period (January–June 2012) at the Department of Nutrition, Pitié-Salpêtrière University Hospital (Paris, France) for management of overweight or obesity. All subjects had a complete work-up including general examination, assessment of body composition by DXA, laboratory tests and TCM examination by one practitioner. Blood sample was collected by nurses and analysed in clinical laboratory in the hospital. The test items were fasting plasma glucose, fasting plasma insulin, total cholesterol, triglycerides, and high density lipoprotein (low density lipoproteins were calculated according to the standard Friedwald equation). All patients signed an informed written consent. The study protocol was approved by the Ethics Committee of Pitié-Salpêtrière Hospital (registration number 1567522).

### 2.2. Inclusion and Exclusion Criteria

Inclusion criteria were male or female patient, aged 18–60 years, with obesity grade I (30 kg/m^2^ ≤ BMI < 35 kg/m^2^) to III (BMI ≥ 40 kg/m^2^) according to WHO classification (WHO, 1997); stable body weight over the last three months (±3 kg); absence of diabetes, hypertension or hypothyroidism (according to history or current treatments), or controlled condition (i.e., HbA1c ≤ 7%, blood pressure in the target range, TSH in the normal range for diabetes, hypertension, and hypothyroidism, resp.). Exclusion criteria included: BMI ≥ 60 kg/m^2^; prior knowledge or practice of TCM; pregnancy or breastfeeding women; serious comorbidities, that is, heart failure, liver disease, renal insufficiency, infection, and neoplasia; severe depression or unstable psychiatric disorder; obesity with a specific cause (e.g., endocrinopathy and genetic syndrome); recent obesity surgery (within 6 months).

### 2.3. Anthropometry

Body weight was measured to the nearest 0.1 kg. Height was measured to the nearest 5 mm, without shoes, with a wall-mounted stadiometer. BMI was calculated as weight divided by the square of height (kg/m^2^). Weight history was assessed by reported weight at age 20 and maximum weight during lifetime.

### 2.4. Body Composition

Body composition was estimated by whole-body fan-beam DXA scanning (Hologic Discovery W, Software v12.6; Hologic, Bedford, MA), as previously described [23]. Body regions (arms, legs, trunk, and head) were delineated with the use of specific anatomical landmarks. Variables from DXA used in analysis were total/trunk/appendicular fat mass (FM, kg) and fat free mass (FFM, kg). FFM was calculated as lean mass (LM, kg) plus bone mineral content (BMC, kg). Appendicular FM (or FFM) was calculated as the sum of arm and leg FM (or FFM). Percent body fat (FM %) was computed as [total FM (kg) divided by body weight (kg)] × 100. FM index (FMi, kg/m^2^) and FFM index (FFMi, kg/m^2^) were computed as total FM divided by height squared and total FFM divided by height squared, respectively, as described elsewhere [24]. Variables used for regional body composition included trunk and appendicular FM (or FFM) in absolute value (kg) or relative to the corresponding body compartment [23]: percent trunk FM (%) was computed as [trunk FM (kg) divided by total FM (kg) × 100], percent appendicular FM (%) was computed as [appendicular FM (kg) divided by total FM (kg) × 100]; similar calculations were performed for percent trunk FFM (%) and percent appendicular FFM (%).

### 2.5. Physical Activity

Physical activity was measured by self-reporting of subject through a lifestyle questionnaire designed by the Department of Nutrition. The questionnaire recorded subject's sedentary time (hours per day) and leisure activity (hours per week). Sedentary occupations among them watching TV, using computer, or reading during working and off-work hours. Physical activities included leisure-time activities (such as brisk walking and gardening) and sports.

### 2.6. ZHENG

According to 〈〈The Internal Medicine of Traditional Chinese Medicine〉〉, we selected total of 15 candidates for ZHENG elements in relation with obesity [25]. “QiXu” (Qi deficiency), “XueXu” (blood deficiency), “YinXu” (Yin deficiency), “YangXu” (Yang deficiency), “QiZhi” (Qi stagnation), “TanShi” (phlegm-dampness obstruction), “Re” (interior heat), and “YuXue” (blood stasis) were 8 candidates for ZHENG essentials. “Xin” (heart), “Gan” (liver), “Pi” (spleen), “Wei” (stomach), “Fei” (lung), “Chang” (colon), and “Shen” (kidney) were 7 candidates for ZHENG locations. One skilled investigator and one 10-year experienced TCM practitioner performed all TCM examinations and completed the TCM syndrome element questionnaire [26]. The examination included assessment of symptoms, signs, tongue examination (photo), and pulse measurement. ZHENG elements were identified by the TCM practitioner according to criteria from 〈〈Syndrome Element Differentiation〉〉. For each subject, he calculated a total symptom score for each ZHENG candidate element according to syndrome scale. If the symptom score was over a threshold of 20, the relevant ZHENG element was identified [26]. All identified elements were used to assess primary ZHENG by means of cluster analysis (see Statistical Analyses). Subjects were assigned to the corresponding primary ZHENG category or cluster according to the statistical output. This assignment was then checked by three experienced TCM practitioners. In case of disagreement, the Delphi method was used to draw a conclusion [27].

### 2.7. Statistical Analyses

Data are presented as means ± s.d. or percent. Normality of distributions was assessed graphically and by the Kolmogorov-Smirnov test. An indicator of insulin resistance, the HOMA2-IR index, was estimated based on fasting glucose and fasting insulin measurements by the updated Homeostasis Model Assessment method [28]. All the identified ZHENG elements were regarded as variables to assess primary ZHENG by method of K-Means cluster analysis. Iterate and classification methods were both used and the maximum iterations were set up at 20. ZHENG essentials and locations were expressed by frequency [occurrence divided by the total number of subjects × 100] or its proportion of total frequency. Chi-square tests were used for comparison. The comparison of obesity phenotypes across primary ZHENG categories was performed by one-way ANOVA. LSD test (or Tamhane's T2 test) was used for post hoc multiple comparisons. All analyses were performed by using PASW Statistics (version 17.0). Significance was judged at *P* < 0.05.

## 3. Results and Discussion

### 3.1. ZHENG Feature

**Figure 1:**
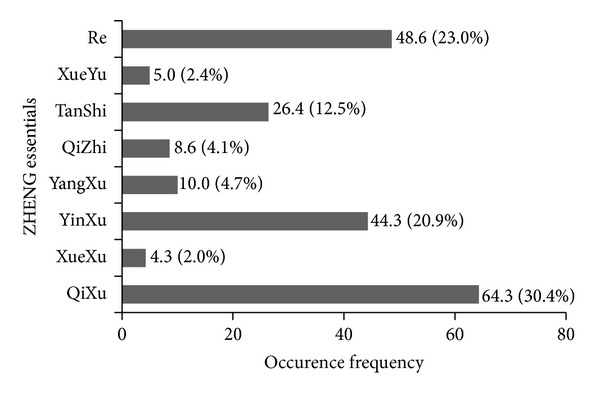
Distribution of ZHENG essentials. Each essential is displayed as frequency (occurrence per 100 patients) and its proportion of total frequency (%). Comparison was performed by using chi-square test (*P* = 0.001).

**Figure 2:**
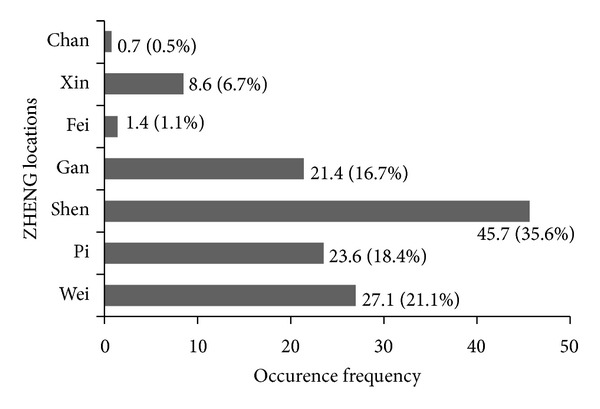
Distribution of ZHENG locations. Each location is displayed as frequency (occurrence per 100 patients) and its proportion of total frequency (%). Comparison was performed by using chi-square test (*P* = 0.001).

In 140 consecutive obese patients examined during the study period (84% women, 40.3 ± 10.3 y, BMI: 39.9 ± 5.8 kg/m^2^), “QiXu,” “Re,” “YinXu,” and “TanShi” were the main ZHENG essentials (total proportion of 86.8%). “Shen,” “Wei,” “Pi,” and “Gan” were the main ZHENG locations (total proportion of 91.8%) (Figures 1 and 2).

After performing cluster analysis, we identified 4 types of primary ZHENG. Each type was named according to its main essentials and locations based on frequency. Type A (“Shen-QiXu-YinXu”) included 52 subjects, its main components were “QiXu” (57.7 frequency), “YinXu” (63.5 frequency), and “Shen” (90.4 frequency). Type B (“Pi-QiXu-TanShi”) included 23 subjects, its main components were “QiXu” (56.5 frequency), “TanShi” (47.8 frequency), and “Pi” (52.2 frequency). Type C (“Gan-Wei-Re”) included 50 subjects, its main components were “Re” (100 frequency), “Gan” (46 frequency), and “Wei” (56.6 frequency). Type D (“Shen-QiXu-TanShi”) included 15 subjects, its main components were “QiXu” (93.3 frequency), “TanShi” (80.0 frequency), and “Shen” (73.3 frequency).

### 3.2. Primary ZHENG and Obesity Phenotypes

Subjects in type D cluster showed a pattern with more serious obesity (higher BMI and larger proportion of subjects with grade III obesity). They also showed special body composition characteristics (increased total FM, FMi, trunk FM, and trunk FM %). Furthermore, they spent the least time in leisure activity among all subjects (Table 1).

Subjects in type B cluster showed different weight history (they were heavier when 20 years old) and had a greater proportion of subjects with obesity grade II. They also differed in regional body composition (reduced trunk FM% and FFM%, elevated appendicular FM %) (Table 1).

Subjects in type A cluster showed reduced appendicular FFM in contrast to those in types B and C. They spent more time in leisure activity. Plasma HDL was reduced in subjects from type C cluster by comparison with other clusters (Table 1).

Regarding a possible effect of age, there was no significant correlation between age and the DXA-assessed body composition parameters. Moreover, comparisons between ZHENG clusters in a restricted sample of younger subjects (age under 40) showed similar results (Tables 2 and 3).

## 4. Discussion

Our study found 4 main ZHENG essentials with a total constituent ratio of 86.8% (“QiXu” 30.4%, “Re” 23.0%, “YinXu” 20.9%, and “TanShi” 12.5%). In addition, we also found 4 main ZHENG locations with a total constituent ratio of 91.8% (“Shen” 35.6%, “Wei” 21.1%, “Pi” 18.4%, and “Gan” 16.7%). “QiXu” and “Shen” showed the highest incidence in ZHENG essentials and locations, respectively. These findings differ from previous results of Chinese research reports [20, 29]. In these studies in Chinese obese patients, “TanShi,” “QiXu,” and “YangXu” were the main ZHENG essentials with “TanShi” being the most frequent. Meanwhile, the main locations were the same as in our study, although “Pi” appeared most involved.

In TCM, ZHENG is presented by combination of essentials and locations. Each patient shows his/her own ZHENG characteristic profile. However, at the level of population with same disease, we may find its common features and major presentations. That is what has been called primary ZHENG. Here it refers to “bianzheng fenxing” (in Chinese). It means major and common combination of ZHENG elements for one disease. Sometimes it is equal to ZHENG presentation for one patient; sometimes it represents main part of ZHENG for one patient. In order to explore primary ZHENG, we need mathematical methods. Among statistic models, the cluster analysis we used appears as an appropriate approach to access primary ZHENG, as shown in recent research [30, 31]. In our study in French obese patients, we identified four types of primary ZHENG by cluster analysis: type A (“Shen-QiXu-YinXu”), type B (“Pi-QiXu-TanShi”), type C (“Gan-Wei-Re”), and type D (“Shen-QiXu-TanShi”). In terms of composition, types A and C shared more proportions than other two types (37.1% and 35.7% versus 16.4% and 10.7%). These findings differ from those found in Chinese obese patients, where the most frequent patterns were “Pi-QiXu-TanShi,” “Wei-Re-TanShi,” “Gan-QiZhi,” “Shen-YinXu-Re,” and “Pi-Shen-YangXu” [29]. Insides, “Gan-Wei-Re” shares major proportion (almost half percent), supported by one 2518-subject survey [32].

Why did these differences happen? It may be due to ethnic differences. Selection bias may be another reason. As the cut-off points for obesity in Chinese population (BMI = 28) is lower than that in the European (BMI = 30), the average BMI of obese subjects in Chinese studies is generally lower than that found in European studies. In our study, the average BMI was almost 40 kg/m^2^. However, the difference of ZHENG feature is not the focus of our study. The discovery of ZHENG feature is the precondition to correlate ZHENG to body composition and other obesity phenotypes, which also supports our point that ZHENG is a candidate for obesity phenotype, even in French obese population.

Among all types of primary ZHENG, type D (“Shen-QiXu-TanShi”) shows increased BMI (43.6 ± 6.6 kg/m^2^ versus 39.7 ± 5.9 kg/m^2^, 38.6 ± 5.7 kg/m^2^, and 39.5 ± 5.2 kg/m^2^, *P* = 0.053), total FM (58.1 ± 11.5 kg versus 49.2 ± 11.1 kg, 49.2 ± 10.4 kg, and 50.6 ± 10.1 kg, *P* = 0.043), FMi (21.4 ± 4.4 kg/m^2^ versus 18.6 ± 4.2 kg/m^2^, 17.6 ± 4.5 kg/m^2^, and 18.0 ± 3.7 kg/m^2^, *P* = 0.029), trunk FM (29.4 ± 6.9 kg versus 24.7 ± 6.4 kg, 23.2 ± 6.1 kg, and 25.7 ± 5.4 kg, *P* = 0.026) and approximately increased weight (118.0 ± 19.6 kg versus 105.4 ± 18.4 kg, 109.3 ± 18.3 kg, and 112.7 ± 18.6 kg, *P* = 0.077). Those subjects appear heavier and fatter and with more central obesity, therefore, presenting more with an “apple” body pattern. However, they had comparable levels of fasting plasma glucose, fasting plasma insulin, blood lipid, and insulin resistance (HOMA2-IR) by comparison to those with other types of primary ZHENG. Apart from type D, type A, type B, and type C showed similar BMI, total FM and FM% (46.5 ± 4.8%, 45.0 ± 6.5%, and 45.2 ± 5.6%), FMi and FFMi (20.8 ± 2.3 kg/m^2^, 20.9 ± 2.7 kg/m^2^, and 21.5 ± 2.9 kg/m^2^). But type B (“Pi-QiXu-TanShi”) shows interesting features in regional body composition. By comparison to other types, trunk FFM% (46.9 ± 4.1% versus 50.2 ± 5.9% and 50.6 ± 4.2%, *P* < 0.05) and trunk FM% (47.0 ± 3.3% versus 48.8 ± 3.3% and 48.2 ± 2.2%, *P* < 0.05) were decreased in type B. In contrast, appendicular FM% was increased in same type (50.3 ± 4.0% versus 47.2 ± 5.9% and 46.6 ± 4.4%, *P* < 0.05). These findings from subjects of type B and type D indicate that we can correlate ZHENG and body composition.

We had performed a literature search through PubMed and Wanfang Data (one principal literature database in China) using the following key words (in English and in Chinese): obesity, body composition, Traditional Chinese Medicine, ZHENG, syndrome differentiation, and pattern differentiation. Few ZHENG-related publications paid attention to body fat or body composition, especially when measured by DXA [10, 33]. Some research related ZHENG to fatty liver, which can be regarded as a special “ectopic” adipose depot. Primary ZHENGs of “Gan-TanShi-Re,” “Gan-Pi-QiXu-TanShi,” and “Gan-Pi-QiZhi” were closely related to fatty liver, based on a survey in 180 overweight and 548 obese subjects, whose BMI ranged from 25 to 40 kg/m^2^ [31]. Because of lack of data, it is difficult to compare our results with previous research. However, if we combine results from subjects with type B and type D, it appears that the unique difference between them is ZHENG location. It might imply to us that the location “Shen” is probably related to total body fat and central obesity, while the location “Pi” is related to appendicular obesity. We need further study to verify this hypothesis.

Apart from body composition, ZHENG also showed associations with other obesity phenotypes. Although type D had increased body weight compared to the three other types, type B showed a different obesity history with highest weight at age 20 (84.2 ± 16.9 kg versus 71.2 ± 21.6 kg, 77.8 ± 15.7 kg, and 71.7 ± 17.0 kg, *P* = 0.039). Type A subjects were found more physically active during leisure activities, while type D spent the least time in leisure activities. Type C had decreased plasma HDL levels (0.41 ± 0.13 mmol/L versus 0.47 ± 0.15 mmol/L, 0.50 ± 0.12 mmol/L, and 0.52 ± 0.15 mmol/L, *P* = 0.019). These findings are dispersive and there was no similar research published previously. Thus, we cannot temporarily presume any conclusion and need further work to verify them. As a pilot study, there are inherent limitations such as the imbalance in sex ratio (woman 84%) and unsymmetrical group assignment. This may explain as well why some findings in our study seem disperse. Nevertheless, we provide evidence that ZHENG shows certain associations with body composition, which clearly deserve further investigation.

## 5. Conclusions

Obesity phenotypes based on body composition differ according to ZHENG in obese patients. This result supports the hypothesis that ZHENG is associated with body composition. Our study is a first step to better understand the contribution of TCM ZHENG to obesity phenotyping. These findings may have implications for patient management.

## Figures and Tables

**Table 1:** Primary ZHENG and obesity phenotype.

	Shen-QiXu-YinXu type A	Pi-QiXu-TanShi type B	Gan Wei Re type C	Shen-QiXu-TanShi type D	*P* value
Age (year)	43.0 ± 10.3^A^	35.6 ± 9.1^B^	38.1 ± 9.7^B^	45.5 ± 9.7^A^	0.002
Sex: female, *n* (%)	47 (90.4)	18 (78.3)	39 (78.0)	14 (93.3)	0.193
Height (m)	1.63 ± 0.08^A^	1.68 ± 0.10^B^	1.69 ± 0.10^B^	1.64 ± 0.07^AB^	0.005
BMI (kg/m^2^)	39.7 ± 5.9^A^	38.6 ± 5.7^A^	39.5 ± 5.2^A^	43.6 ± 6.6^B^	0.053
Grade I, *n* (%)	10 (19.2)	3 (13.0)	11 (22.0)	2 (13.3)	0.019
Grade II, *n* (%)	19 (36.5)	14 (60.9)	17 (34.0)	1 (6.7)	
Grade III, *n* (%)	23 (44.3)	6 (26.1)	22 (44.0)	12 (80.0)	
Weight (kg)	105.4 ± 18.4	109.3 ± 18.3	112.7 ± 18.6	118.0 ± 19.6	0.077
Weighted at the age of 20	71.2 ± 21.6^A^	84.2 ± 16.9^B^	77.8 ± 15.7^AB^	71.7 ± 17.0^A^	0.039
Max weight	115.2 ± 21.3	124.3 ± 27.1	116.8 ± 18.7	123.7 ± 24.1	0.269
Body composition					
Total FM (kg)	49.2 ± 11.1^A^	49.2 ± 10.4^A^	50.6 ± 10.1^A^	58.1 ± 11.5^B^	0.043
FM% (%)	46.5 ± 4.8	45.0 ± 6.5	45.2 ± 5.6	48.5 ± 4.0	0.160
FMi (kg/m^2^)	18.6 ± 4.2^A^	17.6 ± 4.5^A^	18.0 ± 3.7^A^	21.4 ± 4.4^B^	0.029
FFM (kg)	55.4 ± 9.3^A^	59.9 ± 12.9^AB^	61.4 ± 12.5^B^	60.1 ± 9.3^AB^	0.052
FFMi (kg/m^2^)	20.8 ± 2.3	20.9 ± 2.7	21.5 ± 2.9	22.1 ± 2.6	0.312
Trunk FM (kg)	24.7 ± 6.4^A^	23.2 ± 6.1^A^	25.7 ± 5.4^A^	29.4 ± 6.9^B^	0.026
Trunk FM% (%)	48.8 ± 3.3^A^	47.0 ± 3.3^B^	48.2 ± 2.2^A^	49.2 ± 2.2^A^	0.047
Trunk FFM(kg)	27.0 ± 4.8	28.0 ± 5.3	29.5 ± 5.9	29.9 ± 4.8	0.079
Trunk FFM% (%)	50.2 ± 5.9^A^	46.9 ± 4.1^B^	50.6 ± 4.2^A^	50.3 ± 3.8^A^	0.029
Appendicular FM (kg)	23.2 ± 6.1	24.6 ± 4.9	23.7 ± 5.5	27.5 ± 5.1	0.083
Appendicular FM% (%)	47.2 ± 5.9^A^	50.3 ± 4.0^B^	46.6 ± 4.4^A^	47.5 ± 3.6^A^	0.035
Appendicular FFM (kg)	24.2 ± 5.0^A^	27.5 ± 7.6^B^	27.5 ± 6.6^B^	26.0 ± 5.2^AB^	0.042
Appendicular FFM% (%)	43.6 ± 3.3	45.4 ± 3.7	44.5 ± 2.5	43.2 ± 3.8	0.077
Physical activity					
ST (h/day)	7.2 ± 3.2	7.5 ± 3.2	7.9 ± 3.4	8.2 ± 3.1	0.656
LA (h/week)	10.5 (6.0, 20.0)^A^	4.5 (2.2, 9.3)^B^	5.0 (2.5, 11.1)^B^	1.95 (0, 7.3)^C^	0.001
Biologic phenotype					
FPG (mmol/L)	5.2 ± 1.0	5.0 ± 0.9	5.5 ± 1.4	5.8 ± 1.2	0.137
FINS (mUI/L)	16.4 ± 12.5	12.5 ± 7.4	19.8 ± 14.6	20.1 ± 11.5	0.524
HOMA2-IR	2.1 ± 1.6	1.6 ± 1.0	2.5 ± 1.7	2.6 ± 1.5	0.526
TC (mg/dL)	191 ± 42	182 ± 43	192 ± 41	192 ± 40	0.814
TG (mg/dL)	132 ± 95	102 ± 49	132 ± 70	119 ± 146	0.396
HDL (mg/dL)	47 ± 15^A^	50 ± 12^A^	41 ± 13^B^	52 ± 15^A^	0.019
LDL (mg/dL)	119 ± 36	112 ± 37	125 ± 36	117 ± 32	0.523

Values are displayed as means ± s.d. number (percent) or median (percentile 25, percentile 75). For normalized data, comparisons between groups were performed using one-way ANOVA followed by LSD test when applicable. Different superscripts (in uppercase) denote values that are significantly different (*P* < 0.05) from one to the other with post hoc tests. For nonnormalized data, comparisons between groups were performed using Kruskal-Wallis test. Comparisons between age interval, sex, or obesity category were performed using chi-test or R∗C crosstab test.

BMI: body mass index; FM: fat mass; FFM: fat free mass; FMi: fat mass index; FFMi: fat free mass index; obesity grade: I, BMI 30.0–34.9; II, BMI 35.0–39.9; III, BMI ≥40.0 (WHO, 1997); ST: sedentary time; LA: leisure activity; FPG: fasting plasma glucose; FINS: fasting plasma insulin; HOMA2-IR: insulin resistance index of Homeostasis Model Assessment updated; TC: total cholesterol; TG: triglyceride; HDL: high density lipoprotein; LDL: low density lipoprotein.

**Table 2:** Correlation between age and body composition.

Spearman's rho		Weight	Total FM	FMi	FFM	FFMi	Trunk FM	Trunk FFM	Appendicular FM	Appendicular FFM
Age	Correlation	−0.057	−0.007	−0.081	−0.054	0.025	0.040	0.033	−0.174*	−0.090
	*P* value (2-tailed)	0.504	0.933	0.351	0.532	0.774	0.643	0.708	0.043	0.302

Spearman test was used to check the relationship between age and body composition phenotype.

FM: fat mass; FFM: fat free mass; FMi: fat mass index; FFMi: fat free mass index.

*Refers to correlation is significant at the 0.05 level (2-tailed).

**Table 3:** Primary ZHENG and obesity phenotype (age ≤ 40 years).

	Shen-QiXu-YinXu type A	Pi QiXu TanShi type B	Gan Wei Re type C	Shen QiXu TanShi type D	*P* value
Age (year)	34.4 ± 5.0	30.8 ± 4.7	30.8 ± 4.8	35.2 ± 6.5	—
BMI (kg/m^2^)	38.8 ± 4.3	38.6 ± 4.9	40.2 ± 4.8	45.8 ± 8.3	0.027
Weight (kg)	105.5 ± 16.4	111.9 ± 18.7	109.9 ± 15.9	125.9 ± 12.3	0.108
Weighted at the age of 20	77.4 ± 22.7	92.3 ± 12.5	80.4 ± 17.4	87.0 ± 21.1	0.119
Max weight	115.6 ± 21.1	123.8 ± 22.2	114.9 ± 16.6	133.4 ± 34.7	0.188
Body composition					
Total FM (kg)	48.6 ± 9.2	49.8 ± 9.0	51.9 ± 8.3	60.6 ± 12.1	0.053
FM% (%)	45.9 ± 4.3	44.6 ± 7.0	47.3 ± 3.8	48.1 ± 4.6	0.292
FMi (kg/m^2^)	17.9 ± 3.0	17.4 ± 3.8	19.0 ± 2.9	22.2 ± 5.4	0.030
FFM (kg)	56.2 ± 9.1	62.3 ± 14.7	57.6 ± 10.2	63.4 ± 11.8	0.258
FFMi (kg/m^2^)	20.6 ± 2.1	21.2 ± 3.0	21.0 ± 2.9	22.9 ± 3.2	0.387
Trunk FM (kg)	24.0 ± 5.2	23.2 ± 5.5	25.4 ± 4.5	30.5 ± 9.1	0.054
Trunk FM% (%)	48.1 ± 2.9	46.3 ± 3.2	48.3 ± 2.0	49.2 ± 2.8	0.061
Trunk FFM (kg)	27.0 ± 4.7	28.6 ± 6.1	27.9 ± 5.3	31.2 ± 6.0	0.408
Trunk FFM% (%)	49.4 ± 4.5	46.3 ± 4.2	48.7 ± 3.8	49.5 ± 6.1	0.165
Appendicular FM (kg)	23.3 ± 5.0	25.2 ± 4/3	25.3 ± 4.8	28.9 ± 3.6	0.090
Appendicular FM% (%)	48.0 ± 4.7	50.9 ± 4.0	48.7 ± 3.7	48.4 ± 5.8	0.212
Appendicular FFM (kg)	25.0 ± 4.8	29.1 ± 8.7	25.4 ± 4.8	28.1 ± 6.0	0.131
Appendicular FFM% (%)	44.3 ± 3.1	46.1 ± 3.8	44.0 ± 1.8	44.1 ± 3.0	0.152
Physical activity					
ST (h/day)	6.9 ± 2.7	7.7 ± 2.8	8.7 ± 3.6	10.3 ± 3.2	0.113
LA (h/week)	9.9 (6.2, 16.0)	4.5 (2.0, 10.5)	5.3 (1.9, 11.1)	6.1 (1.4, 7.7)	0.059

Values are displayed as means ± s.d. number (percent) or median (percentile 25, percentile 75). For normalized data, comparisons between groups were performed using one-way ANOVA followed by LSD test when applicable. For nonnormalized data, comparisons between groups were performed using Kruskal-Wallis test.

BMI: body mass index; FM: fat mass; FFM: fat free mass; FMi: fat mass index; FFMi: fat free mass index; ST: sedentary time; LA: leisure activity.
